# Endocrine disruption in aquatic systems: up‐scaling research to address ecological consequences

**DOI:** 10.1111/brv.12360

**Published:** 2017-08-09

**Authors:** Fredric M. Windsor, Steve J. Ormerod, Charles R. Tyler

**Affiliations:** ^1^ School of Biosciences Cardiff University Cardiff CF10 3AX U.K.; ^2^ Department of Biosciences University of Exeter Exeter EX4 4PS U.K.

**Keywords:** aquatic pollution, ecotoxicology, endocrine‐disrupting chemicals, food webs, populations

## Abstract

Endocrine‐disrupting chemicals (EDCs) can alter biological function in organisms at environmentally relevant concentrations and are a significant threat to aquatic biodiversity, but there is little understanding of exposure consequences for populations, communities and ecosystems. The pervasive nature of EDCs within aquatic environments and their multiple sub‐lethal effects make assessments of their impact especially important but also highly challenging. Herein, we review the data on EDC effects in aquatic systems focusing on studies assessing populations and ecosystems, and including how biotic and abiotic processes may affect, and be affected by, responses to EDCs. Recent research indicates a significant influence of behavioural responses (e.g. enhancing feeding rates), transgenerational effects and trophic cascades in the ecological consequences of EDC exposure. In addition, interactions between EDCs and other chemical, physical and biological factors generate uncertainty in our understanding of the ecological effects of EDCs within aquatic ecosystems. We illustrate how effect thresholds for EDCs generated from individual‐based experimental bioassays of the types commonly applied using chemical test guidelines [e.g. Organisation for Economic Co‐operation and Development (OECD)] may not necessarily reflect the hazards associated with endocrine disruption. We argue that improved risk assessment for EDCs in aquatic ecosystems urgently requires more ecologically oriented research as well as field‐based assessments at population‐, community‐ and food‐web levels.

## I. INTRODUCTION

Endocrine‐disrupting chemicals (EDCs) remain an active topic in contemporary ecotoxicology due to their proven environmental impacts (Zhou, Cai & Zhu, 2010; Wang & Zhou, 2013) and postulated health effects (Kabir, Rahman & Rahman, 2015). Over the past decade published work on EDCs has provided a strong mechanistic understanding of exposure effects (Colborn, vom Saal & Soto, 1993; Tyler, Jobling & Sumpter, 1998; Kloas *et al*., 2009; Orton & Tyler, 2012; Söffker & Tyler, 2012; Tijani, Fatoba & Petrik, 2013). Far less consideration, however, has been given to processes and interactions controlling the effects of EDCs at broader ecological scales, including inter‐ and intra‐specific interactions within populations and food webs (Segner, 2011; Brodin *et al*., 2014; Schoenfuss *et al*., 2015). Understanding the effects of EDCs on processes operating at these broader scales is essential, but also challenging, because their effects can be pervasive and they are generally sub‐lethal in nature. Although EDCs can induce deleterious effects in a wide range of organisms across different trophic levels (Brander, 2013), there is insufficient knowledge for environmental regulators to assess the impacts and risks posed by EDC pollution to populations, communities and ecosystems (e.g. Mills & Chichester, 2005; Hallgren *et al*., 2012).

Herein, we evaluate critically the known and potential effects of EDCs on natural ecological systems. We highlight a need for EDC research to incorporate processes and effects at broader spatial and temporal scales, illustrating how such studies have helped to advance our understanding of EDC impacts beyond common approaches to EDC testing. We also suggest an integrated research strategy for EDCs that develops previous designs from other pollutants to generate more environmentally relevant data. Finally, we consider further research needs to understand better the effects of EDCs on natural systems.

## II. THE BENEFITS OF UP‐SCALING EDC RESEARCH

**Figure 1:**
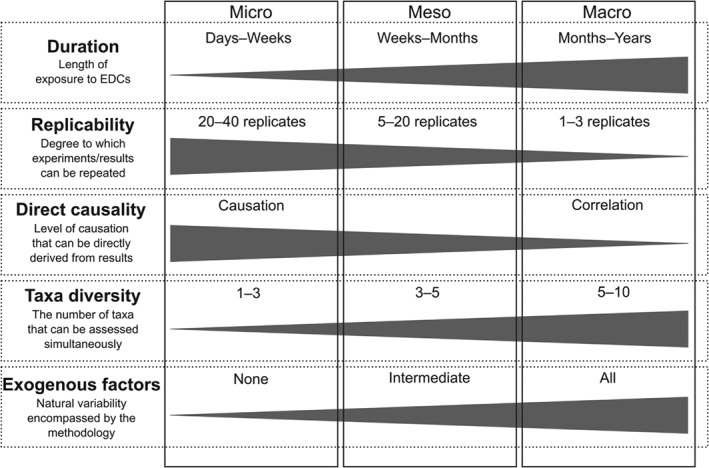
Conceptual differences in endocrine‐disrupting chemical (EDC) experimental framework design and expected outcomes of micro‐, meso‐ and macroscale assessments.

The requirement for information on population effects of EDC exposure to inform ecological risk assessments has led to the extrapolation of individual‐based experimental bioassays (e.g. Jobling *et al*., 2002b; Miller & Ankley, 2004; Gutjahr‐Gobell *et al*., 2006; Lange *et al*., 2008; Brander *et al*., 2016). Such extrapolations assume, however, that the effects of EDC exposure within individual‐based bioassays generally show simple, direct and invariant relationships with impacts on populations and communities, even if safety factors are used to account for uncertainties associated with these extrapolations. Assessments involving wild populations, however, demonstrate discontinuities between the results of individual‐ and population‐level assessments (Jobling *et al*., 2002a; Brown *et al*., 2005; Lange *et al*., 2011; Hamilton *et al*., 2014). Fundamental differences in the ecological processes represented within micro‐, meso‐ and macroscale assessments (Fig. 1) are potentially responsible for this disparity. Specifically, these differences include the nature of the EDC exposure regime, possible compounding environmental influences (e.g. multiple stressors), and the fact that multiple effect mechanisms may operate through trophic interactions across food webs at the macroscale (Hamilton *et al*., 2016a). There are several potential inconsistencies in findings about endocrine disruption from different biological, spatial and temporal scales. For example, cause–effect relationships reflect the methods used and scales at which studies are completed and this creates a challenge in determining mechanistic relationships and emergent effects at broader spatio‐temporal extents. As an example, feminisation at the individual level would suggest significant potential population effects, but studies at broader spatial scales have indicated that population‐level effects depend on mating‐system dynamics (White *et al*., 2017). On the one hand, the low cost of sperm production relative to eggs means that males are able to fertilise multiple females, thus the feminisation of males may have little effect on population dynamics (White *et al*., 2017). On the other hand, mating systems may prevent male promiscuity, meaning that feminisation and minor alterations in the sex ratio result in negative effects on populations (White *et al*., 2017). Currently, little consideration is generally given to natural complexity in ecological and toxicological processes within experimental research designs (see Barton, 2003). Models developed for up‐scaling from individual‐based assessments to population scales are therefore inherently weak, and may even be flawed, as they provide limited appreciations of wider controls on higher levels of biological organisation. Factors such as density‐dependence, adaptation, trophic interactions, likelihood of population exposure (habitat preferences), as well as species‐specific life‐history traits of organisms, are all likely to have a significant impact on endocrine disruption, yet none of these characteristics are considered in common experimental assessments used to investigate the ecological impacts of EDC exposure.

Research that considers processes over longer periods of time (e.g. entire life cycles) and at higher levels of biological organisation (e.g. populations and food webs) overcomes several limitations associated with most current experimental ecotoxicology bioassays (Geiszinger *et al*., 2009). The complexity associated with analysis of mesocosm and field scenarios, however, has restricted the uptake of these research designs. Furthermore, many field studies are characterised by correlation and weak inference in comparison to well‐established mechanistic knowledge developed under more‐controlled experimental conditions. A combination of experimental and field‐based studies across a range of ecological scales is thus required for an improved understanding of population‐ and food‐web‐level responses to EDC exposure. This approach has, however, had relatively little uptake (Patiño & Carr, 2015) and studies assessing the effects of EDCs at community and food‐web scales remain scarce (Boxall *et al*., 2012). Contemporary studies have consequently called for a greater focus on broader scale ecological and toxicological processes (Brodin *et al*., 2014; Kidd *et al*., 2014).

## III. ADVANCES IN BROAD‐SCALE EDC RESEARCH

Here, we assess critically recent findings derived from EDC research focusing on processes operating at broad spatial and temporal scales and highlight the limitations associated with using experimental bioassays conducted without due consideration of natural system dynamics. This builds upon previous conceptual reviews of the role of theoretical ecology in enhancing ecotoxicological studies (e.g. Relyea & Hoverman, 2006).

### (1) Biotic interactions and trophic transfer of EDCs through food webs

The effects derived from EDC exposure within natural systems are variable and influenced by biological processes including competitive interactions and predation. Only a few examples exist regarding how biotic factors affect the severity of endocrine disruption, but a suite of processes appear to provide an important influence on the risk associated with EDC exposure within ecosystems. The behaviour of organisms in response to EDC exposure, in particular, can result in important ecological effects and in some cases, behavioural changes enhance adverse effects of EDC exposure (Melvin & Wilson, 2013). As well as providing the potential to exacerbate an effect at higher levels of biological organisation, interactions among individuals can also buffer the observed effects of EDC exposure. An example of this is density‐dependent compensatory effects in zebrafish *Danio rerio* (Hamilton) populations that have been shown to alleviate negative individual reproductive effects of octylphenol exposure (Hazlerigg *et al*., 2014). Effects such as those detailed above are rarely considered or captured in laboratory‐based studies and the consequences of these alterations could exacerbate the effects of EDCs at higher levels of biological organisation and within natural systems.

Biotic and abiotic processes can influence the trophic transfer of EDCs within aquatic ecosystems. Alkylphenols, pyrethroids, polychlorinated biphenyls (PCBs), polybrominated diphenyl ethers (PBDEs) and diclofenac have been shown to partition, accumulate and magnify within components of aquatic food webs (see Table 1) and exhibit different entry and transfer pathways within the environment (Burreau *et al*., 1997, 2006; Correa‐Reyes *et al*., 2007; Corcellas, Eljarrat & Barcelo, 2015; Muggelberg *et al*., 2017). Many EDCs are hydrophobic in nature and readily partition out of the water column through adsorption to both suspended and benthic sediments (Petrović *et al*., 2001). Consequently, a significant proportion of the total pollutant load entering aquatic food webs is likely to be through benthic taxa interacting with sediments (e.g. sediment ingestors) (Brooks, Gaskell & Maltby, 2009; Wu *et al*., 2009). Dietary transfers, however, are not the main route of uptake for many EDCs, and for selected compounds (e.g. carbamazepine and diphenhydramine) direct adsorption from the water column is a major route for their bioaccumulation (Du *et al*., 2014, 2015, 2016). This transfer of EDCs directly from the water column into aquatic organisms can occur either through passive adsorption, whereby the skin and respiratory surfaces enable diffusion or *via* assimilation of EDCs adhering to suspended organic matter (Zhou *et al*., 2007). In natural systems, it is likely that most EDCs enter organisms by multiple uptake pathways. Thus, EDC exposure within natural systems may be intermittent, as in dietary intake, or possibly continuous *via* the water column.

**Table 1:** Bioaccumulation factors (BAFs) for endocrine‐disrupting chemicals (EDCs) in aquatic organisms

Chemical group	Compound	log K_OW_	log BCF/BAF	Approximate trophic level	Organism	Source
Organobromines	BDE‐100	7.24	7.50	3	*Salvelinus namaycush*	Streets *et al*. (2006)
	BDE‐47	6.81	7.30	3	*Salvelinus namaycush*	
	BDE‐66	—	7.30	3	*Salvelinus namaycush*	
	BDE‐99	7.32	6.70	3	*Salvelinus namaycush*	
	γ‐HBCD	5.48	4.51	3	*Carassius auratus*	Wu *et al*. (2011)
	HBB	6.09	3.48	2	*Cipangopaludina chinensis*	
		6.09	4.47	3	*Carassius auratus*	
	PBDEs	6.27	0.96	2	*Gammarus pulex*	Tlili *et al*. (2012)
		6.27	0.79	2	*Echinogammarus stammers*	Viganò *et al*. (2009)
Organochlorines	DDE	6.51	1.65	3	*Rana* spp.	Albanis *et al*. (1996)
		6.51	2.40	5	*Egretta garzetta*	
	DDT	6.52	4.00	2	*Pomacea* spp.	Siriwong *et al*. (2009)
		6.52	4.40	2	*Macrobranchium lanchesteri*	
		6.52	6.60	2	*Filopaludina mertensi*	
	HCB	5.72	6.20	2	*Tubifex tubifex*	Egeler *et al*. (1997)
		5.72	2.00	2	*Eisenia fetida/andrei*	
	Lindane	3.80	2.20	3	*Rana* spp.	Albanis *et al*. (1996)
		3.80	2.35	5	*Egretta garzetta*	
		3.80	4.40	2	*Tubifex tubifex*	Egeler *et al*. (1997)
		3.80	2.50	2	*Eisenia fetida/andrei*	
	PCBs	6.50	7.63	3	*Perca fluviatalis*	Bremle *et al*. (1995)
		6.50	6.60	1	*Selenastrum* spp.	Stange & Swackhamer (1994)
		6.50	6.10	1	*Anabaena* spp.	
Organophosphates	Chlorpyrifos	4.96	5.99	2	*Mytilus galloprovincalis*	Serrano *et al*. (1997)
	Methidathion	2.42	5.26	2	*Mytilus galloprovincalis*	
	TrBT	9.49	3.37	2	*Ancylus fluviatalis*	Ruhí *et al*. (2015)
		9.49	3.61	2	*Hydropsyche* spp.	
		9.49	3.53	3	*Phagocata vitta*	
Pharmaceuticals	Carbamazepine	2.25	3.03	3	*Oreochromis niloticus*	Garcia *et al*. (2012)
	Diclofenac	4.01	0.92	3	*Oncorhynchus mykiss*	Fick *et al*. (2010)
		1.90	6.86	3	*Hemiculter leucisculus*	Liu *et al*. (2015b)
	Dilitiazem	2.70	3.18	3	*Oncorhynchus mykiss*	Fick *et al*. (2010)
	Diphenhydramine	3.11	2.77	3	*Gambusia holbrooki*	Wang & Gardinali (2013)
	Erythromycin	3.16	5.67	2	*Planorbidae* spp.	Du *et al*. (2015)
	Gemfibrozil	4.77	4.73	3	*Gambusia holbrooki*	Mimeault *et al*. (2005)
	Ibuprofen	3.79	4.06	3	*Oncorhynchus mykiss*	Fick *et al*. (2010)
	Oxazepam	2.24	0.30	2	*Coenagrion hastulatum*	Brodin *et al*. (2014)
	Propranolol	3.48	8.29	3	*Hemiculter leucisculus*	Liu *et al*. (2015b)
	Roxithromycin	2.75	8.87	3	*Hemiculter leucisculus*	
Phenols	BPA	3.40	4.97	2	*Pisidium amnicum*	Heinonen *et al*. (2002)
		3.40	8.48	1	Benthic algae	Yang *et al*. (2014)
	Nonylphenol	4.48	8.85	1	*Isochyrysis galbana*	Correa‐Reyes *et al*. (2007)
		4.48	2.64	2	*Lumbriculus variegatus*	Mäenpää & Kukkonen (2006)
	NPEO2	4.20	3.14	1	*Cladophora glomerata*	Ahel *et al*. (1993) and Staples *et al*. (1998)
		4.20	−0.22	3	*Oncorhynchus mykiss*	
Pyrethroids	Cypermethrin	5.20	5.74	2	*Chironomus tentans*	Muir *et al*. (1985)
	Deltamethrin	5.20	5.76	2	*Chironomus tentans*	
	Fenvalerate	5.20	4.93	2	*Chironomus tentans*	
	Parathion	3.83	4.62	3	*Gnathopogon caerulescens*	Tsuda *et al*. (1994)
	Permethrin	6.20	5.56	2	*Chironomus tentans*	Muir *et al*. (1985)
	Vamidothion	0.12	6.56	3	*Gnathopogon caerulescens*	Tsuda *et al*. (1994)
Steroidal androgens and oestrogens	4‐AD	—	5.39	2	*Meretrix lusoria*	Liu *et al*. (2015a)
	ADD	—	6.33	2	*Meretrix lusoria*	
	Boldenone	—	8.01	2	*Meretrix lusoria*	
	EE2	4.01	0.80	2	*Chironomus tentans*	Dussault *et al*. (2009)
		4.01	4.23	1	Phytoplankton	Xie *et al*. (2015)
		4.01	4.89	3	*Pelteobagrus fulvidraco*	
	Norgestrel	3.48	6.28	2	*Meretrix lusoria*	Liu *et al*. (2015a)
		3.48	6.14	3	*Lutjanus erythopterus*	
	Progesterone	3.87	7.70	2	*Meretrix lusoria*	
	Testosterone	3.32	8.29	2	*Meretrix lusoria*	

Chemicals are divided into organobromines, organochlorines, organophosphates, pharmaceuticals, steroidal androgens and oestrogens, phenols and pyrethroids. Where replicates or multiple measurements were reported within studies a mean value is presented.

4‐AD, 4‐androstene‐3,17‐dione; ADD, androsta‐1,4‐diene‐3,17‐dione; BDE, brominated diphenyl ether; BPA, bisphenol A; DDT, dichlorodiphenyltrichloroethane; DDE, dichlorodiphenyldichloroethylene; EE2, 17α‐ethinyloestradiol; HBB, hexabromobenzene; HBCD, hexabromocylcododecane; HCB, hexachlorobenzene; NPEO2, nonylphenol ethoxylate 2; PBDE, polybrominated diphenyl ether; PCB, polychlorinated biphenyl; TrBT, tris‐(2‐butoxyethyl)‐phosphate.

BAF, bioaccumulation factor; BCF, bioconcentration factor; Log K_OW_, octanol/water partition coefficient. Log K_OW_ values were taken from https://pubchem.ncbi.nlm.nih.gov/.

Upon entry into organisms the transfer of EDCs within aquatic food webs is affected by a series of biological controls, including the organism's physiology, and *via* biotic interactions. The biological traits of organisms, including functional feeding guilds, influence the bioaccumulation, biomagnification and transfer of EDCs (Muñoz *et al*., 2009; Damásio *et al*., 2011). Bioaccumulation can vary across trophic levels (Ruhí *et al*., 2015), but even within the same trophic level individual biological traits, including size, can influence EDC uptake (Sidney *et al*., 2016). Many organisms exhibit an ability effectively to eliminate selected EDCs from tissues, thereby mitigating their accumulation *via* diet or water and subsequent transfer (Norman *et al*., 2007; Al‐Ansari *et al*., 2013). These assessments demonstrate the importance of biological interactions in the trophic transfer of EDCs within natural systems and indicate why responses may deviate from those expected from experimental, laboratory‐based exposure assessments on individual organisms. Further research is, however, required to understand better the influence of biological traits on the bioaccumulation and ecological risk of EDCs.

Interactions between the direct effects of endocrine disruption and the subsequent transfer of EDCs through ecosystems may also occur, supporting that alterations in individual‐level effects may have consequences for wider biological systems (Brooks *et al*., 2009). A specific illustration of this is provided by Brodin *et al*. (2013, 2014) where an increased feeding rate of perch (*Perca fluviatilis* L.) in a behavioural response to oxazepam exposure resulted in enhanced consumption of its damselfly prey (*Coenagrion hastulatum* Charpentier), and in turn an increase in the transfer and bioaccumulation of oxazepam. These examples illustrate that ecological risks for some EDCs that affect ecosystem processes (e.g. feeding behaviour and bioaccumulation potential) may be greater than commonly appreciated within aquatic ecosystems.

### (2) Adaptation to EDC exposure

Individuals, populations and food webs have varying levels of resilience to environmental stressors (Harrison, 1979), but in most cases organisms in aquatic ecosystems are able to persist at low levels of stress, even in multi‐stressor environments (Vinebrooke *et al*., 2004). There is little field‐based information, however, on the ecological and evolutionary resilience of individuals and populations to endocrine disruption, although the presence of adaptation is widely displayed within experimental assessments (see Wu, Siu & Shin, 2005). Many existing studies do not assess adaptations directly, instead indicating the reduction in effect size over the duration of exposure, which occurs more rapidly for individuals in comparison to populations and communities (Wu *et al*., 2005). Several field studies have identified populations of aquatic organisms resistant to certain EDCs. For example, Weston *et al*. (2013) indicated that point mutations at the pyrethroid target site (voltage‐gated Na^+^ channel) in *Hyalella azteca* (Saussure) populations meant that resistant individuals did not experience the neurotoxic effects observed in non‐resistant populations, instead exhibiting oxidative stress only at considerably higher pyrethroid concentrations. Varying levels of resistance were found across several populations. Adaptation has also been observed within fish assemblages (Hamilton *et al*., 2016b). Both the Atlantic tomcod (*Microgadus tomcod* Walbaum) and the Atlantic killifish (*Fundulus heteroclitus* L.) can adapt to polycyclic aromatic hydrocarbon (PAH) and PCB exposure in natural systems (Clark *et al*., 2010; Wirgin *et al*., 2011), but through different mechanisms. In *M. tomcod* a six‐base deletion in the aryl hydrocarbon receptor 2 (AHR2) restricted inducible gene expression and was responsible for the observed resistance to EDC exposure (Wirgin *et al*., 2011). In comparison, resistance in *F. heteroclitus* individuals was generated by single nucleotide polymorphisms in the regulatory regions of the cytochrome P4501A gene (Clark *et al*., 2010; Reid *et al*., 2016).

Resistance, and/or adaptation has significant implications for the potential broad‐scale effects of endocrine disruption in aquatic systems. A recent example in *H. azteca*, showed that populations pre‐exposed to the pyrethroid pesticide Permethrin were able to persist under higher environmental concentrations (>210 ng l^−1^) than those populations which were not pre‐exposed (Muggelberg *et al*., 2017). This adaptation meant that resistant individuals provided a source of dietary exposure for fathead minnows (*Pimphales promelas* Rafinesque) under conditions within which non‐resistant individuals cannot survive. Within natural systems, adaptation of individuals or populations leads to an enhanced risk of bioaccumulation with increasing concentrations of EDCs. Adaptation to endocrine disruption indicates that organisms may be able to persist at environmentally relevant concentrations of EDCs, yet it also suggests potential for increased flux of EDCs through food webs. Changes in the bioaccumulation and transfer of EDCs potentially lead to increases in the body burden of higher trophic‐level organisms, increasing the likelihood of adverse effects across the aquatic food web.

### (3) Long‐term, life‐cycle and transgenerational EDC effects

There have been relatively few assessments of EDCs for long exposure durations, over full organism life cycles and/or over multiple generations, even though many organisms will be exposed for prolonged periods of time. Chronic exposure studies that have been undertaken have provided several significant advances. Firstly, in most cases they have shown that the effects are greater than for short‐term exposures (Keiter *et al*., 2012; Tassou & Schulz, 2013). Secondly, different health effects have been identified for longer‐term exposures in comparison to short‐term exposures. For example, for 17α‐ethinyloestradiol (EE2) exposure, effects reported on mating behaviour, growth and survival in *D. rerio* individuals differed between exposure periods of 0–21 and 0–75 days post‐fertilisation (Segner *et al*., 2003). Thirdly, unanticipated effects have been identified following chronic exposures to EDCs. Exposure of rainbow trout (*Oncorhynchus mykiss* Walbaum) eggs to an environmental oestrogen, bisphenol A (BPA), over a range of concentrations including 300 and 3000 ng l^−1^ resulted in lower energy levels in larvae to first feeding, reductions in specific growth and restricted food conversion ratios (Birceanu, Servos & Vijayan, 2015). Finally, chronic exposure studies have helped to highlight life‐stage‐specific susceptibilities to the effects of EDCs. Schäfers *et al*. (2007) illustrated that the chronic effects on sexual differentiation in *D. rerio* resulting from lifelong exposure to 10 ng l^−1^ of EE2 were more pervasive than the reversible effects induced by exposure extending over the period of gonadal differentiation only.

It must be emphasised that not all EDC effects are necessarily permanent; some are transient in nature and the organism may recover after the exposure is removed. Examples include the reported partial recovery from the effects of EE2 (5 ng l^−1^) on gonad differentiation in *D. rerio* after a 5‐month depuration period post‐EE2 exposure (Nash *et al*., 2004). Complete recovery of biological function was observed in a full‐life‐cycle analysis of *D. rerio* after exposure to EE2 (3 ng l^−1^) (Fenske *et al*., 2005). Here exposure to EE2 from the fertilised egg stage for 118 days post‐fertilisation inhibited gonad differentiation in males, but a 58‐day post‐exposure period of depuration resulted in resumption and subsequent completion of testicular differentiation. Reproduction in *D. rerio* has also been shown to recover completely after exposure to zearalenone; exposure to 1000 ng l^−1^ zearalenone for 140 days induced a female shift in the population sex ratio, but a subsequent period of depuration for 42 days resulted in recovery of relative fecundity (Schwartz *et al*., 2013). The ability to recover will, in part, depend on EDC exposure concentration and the consequent nature and severity of effect(s). In other studies on *D. rerio*, e.g. Schäfers *et al*. (2007) and Baumann *et al*. (2014), individuals did not show full recovery following EE2 exposure at 9.3 ng l^−1^ or trenbolone (an androgen used as a growth promotor for cattle in the USA) exposure at 30 ng l^−1^. The length of both exposure and period for depuration thus appear to be important in weighing up the potential for biological impacts of EDCs in natural systems. The fact that EDCs can act through multiple pathways means that it is especially difficult to identify chronic and life‐stage‐specific effects (Sohoni & Sumpter, 1998). Pinpointing these effects is further hindered by the fact that effect mechanisms for many EDCs are not well defined. As an example, phthalate esters [e.g. di‐n‐butyl phthalate and di(2‐ethylhexyl)phthalate] have been identified as both oestrogen receptor agonists and androgen receptor antagonists (Takeuchi *et al*., 2005). Furthermore, exposure to these compounds maintains a range of individual‐level effects, including alterations in cellular proliferation, biosynthesis and apoptosis, as well as several immune responses (Milla, Depiereux & Kestemon, 2011; Mankidy *et al*., 2013). Thus, when considering the spatio‐temporal dynamics of EDC pollution within aquatic systems it is important to assess all the effects that may manifest. In natural systems, exposure to EDCs in periodic urban run‐off inputs may result in different effects compared with continuous emissions from wastewater treatment works (WwTWs).

Transgenerational studies on the effects of EDCs further highlight the importance of considering temporal scale in effect analyses. There is a mounting consensus that EDC exposure effects can span multiple generations, and may induce different impacts in offspring compared with the parental generation (Skinner, Manikkam & Guerrero‐Bosagna, 2011; Bhandari, vom Saal & Tillitt, 2015). Some of the adverse effects observed in subsequent generations have been shown not to be induced through the direct modulation of DNA sequences, but rather through permanent alterations in the epigenome promoting transgenerational phenotypes (Skinner *et al*., 2011; Head, 2014). This mechanism can promote transmission of potentially susceptible phenotypes to the offspring of affected organisms, and may enhance the adverse impacts of EDCs within subsequent generations (Sowers *et al*., 2009). Exposure during early life or at particularly susceptible life stages can also have effects that span the lifetime of the affected organism and potentially lead to adverse effects in subsequent generations (Head, 2014). These changes can be through somatic and gametic effect pathways (Faulk & Dolinoy, 2011). Consequently, epigenomic changes resulting from EDC exposure may lead to transgenerational effects, and possibly different population‐level impacts within natural systems because of cumulative adverse effects in multiple generations (Bernal & Jirtle, 2010).

Of note is the fact that contemporary assessments of EDCs in the laboratory are confined to a restricted range of short‐lived species suitable for experiments; for fish, notably *D. rerio*, *P. promelas* and medaka (*Oryzias latipes* Temminck & Schlegel). Whilst these taxa are convenient as study models, they may not necessarily allow the accurate prediction of effects within populations of longer‐lived organisms which may accumulate greater levels of EDCs over longer periods of time and have slower generational turnover, and thus a lower ability to adapt in response to toxicological impacts. Further efforts to understand long‐term exposure effects across a wider range of taxa are urgently required.

### (4) Interactive mixtures of EDCs

Wastewater effluents and other pollutant sources are often composed of highly complex mixtures, and interactions between EDCs and of EDCs with other chemicals could alter their biological effects (Keiter *et al*., 2012; Schoenfuss *et al*., 2015). The potential for additive effects of EDCs and other chemicals is significant. Most experiments on EDCs, however, have assessed only the effects of individual chemicals, with a small number of exceptions (e.g. Thorpe *et al*., 2003; Brian *et al*., 2007). A range of adverse, sub‐lethal impacts may occur that are not always predictable from assessments of individual components (Kortenkamp, 2007; Viñas, Jeng & Watson, 2012) or *via* simple additive‐effect modelling (Silva, Rajapakse & Kortenkamp, 2002). Compounds with dissimilar modes of action may induce novel effects, operating through multiple mechanisms (Viñas *et al*., 2012). Sárria *et al*. (2011) demonstrated that exposure to EE2 and tributyltin (TBT) caused alterations in the behavioural responses of juvenile black‐striped pipefish (*Syngnathus abaster* Risso). TBT depressed the burst‐swimming response known to result from EE2 exposure, whilst EE2 influenced the alterations in the time spent in secluded areas generated by high concentrations of TBT. Consequently, when mixtures of EDCs combine with processes such as competition and predation, a range of complex and often unpredictable effects can result.

There are also reports of a non‐monotonic dose–response relationship resulting from exposure to EDC and their mixtures (Vandenberg *et al*., 2012). Non‐monotonic dose–response relationships are not unique to EDCs, but they have been reported more frequently for EDCs than for other toxicants (Vandenberg, 2014), in part reflecting the use of more sensitive endpoints or the wider range of concentrations tested (vom Saal *et al*., 2010; Vandenberg *et al*., 2013; Vandenberg, 2014). Controversially, it has been proposed that hormesis, where marked beneficial low‐dose effects are observed, may be responsible for the non‐monotonic dose–response relationships (Calabrese, 2005). This conclusion has been disputed, with some arguing that the impacts of oestrogenic EDCs always remain negative irrespective of concentration (Weltje, vom Saal & Oehlmann, 2005). Many examples exist of non‐monotonic dose–response relationships for EDCs with markedly different physicochemical properties. Pyrethroid pesticides, for example, generally exhibit greater negative effects at lower concentrations (Brander *et al*., 2016), and BPA shows a non‐monotonic transcriptional‐effect response (Villeneuve *et al*., 2012). There appears to be a wide range of effects that exhibit non‐monotonic relationships with several EDCs.

The identification of non‐linear, non‐monotonic, and in some cases hormetic, relationships across many studies has led some authors to suggest that effects observed at high EDC concentrations may not represent those at environmentally relevant concentrations or for mixtures of EDCs (Beausoleil *et al*., 2013; Vandenberg, 2014). Thus, the lowest observed effect levels (LOELs) recorded within experimental bioassays may not accurately extrapolate to the lowest concentrations present within natural systems (Vandenberg *et al*., 2012). It has been suggested that alternative relationships (U‐ or inverted U‐shaped) may better reflect effects associated with environmental EDC exposure (Vandenberg *et al*., 2014; Vandenberg & Bowler, 2014; Zoeller & Vandenberg, 2015). This challenges the concentration‐specific understanding of endocrine disruption within natural systems and poses a significant challenge for risk assessment if true (Futran Fuhrman, Tal & Arnon, 2015).

### (5) EDCs within the context of multiple stressors

Accounting for environmental variation is crucial in determining the effects of EDC exposure within natural systems, as multiple covariant environmental variables influence observed effects within natural systems (Daughton, 2004; Damásio *et al*., 2011). Previous assessments have used the statistical and environmental control provided by experimental bioassays to eliminate confounding relationships between influential variables present within natural environments. However, interactions between multiple stressors ultimately dictate the relative severity of EDC exposure and subsequent ecological risk within ecosystems (Hooper *et al*., 2013). Recent research has demonstrated the importance of assessments incorporating and accounting for exogenous environmental characteristics, such as water temperature, physicochemical conditions and biotic interactions. These abiotic and biotic stressors may interact with one another as well as with EDCs to affect the outcome in exposed organisms. A modelling study by An *et al*. (2009) assessing wild roach (*Rutilus rutilus* L.) populations demonstrates the potential for interactive effects of multiple stressors. Here, the feminisation of individuals generated by endocrine disruption appeared to have negligible effects on population extinction risk, yet the combination of exposure and selective fishing practices resulted in significant increases in local population extinction rates. The feminising effect of oestrogenic EDCs in isolation does not always result in significant population effects (see Hamilton *et al*., 2016a) and in some cases the population‐level threats from masculinisation are greater than from feminisation. The relative threat of both feminisation and masculinisation, however, is dependent on the optimal sex ratio of individual populations (White *et al*., 2017). Fish species exhibiting a non‐linear mating function (non‐linear response of reproductive capacity to changing sex ratio) did not exhibit reduced reproductive output when few males were present, however, the overall reproductive output of the population was significantly reduced by declines in the relative abundance of females (White *et al*., 2017).

Studies assessing temperature and EDC exposure indicate that stressor–EDC interactions may take multiple forms, with EDC exposure in some cases driving alterations in the effects of temperature increases (Jenssen, 2006), while in other cases temperature determines the severity of ecological effects derived from EDC exposure (Moe *et al*., 2013). The importance of interactions between two stressors has been relatively well demonstrated by contemporary research, yet these studies are still not representative of the true complexity present within natural systems. More recent research has attempted to encapsulate a greater number of stressors. For example, Brown *et al*. (2015) showed that a combination of EDC exposure, temperature increases and inbreeding led to a significantly skewed sex ratio in *D. rerio* populations. Increases in temperature (28–33°C), clotrimazole exposure (2000 and 10000 ng l^−1^) and inbreeding together had an additive effect, with a marked increase in the male‐skew of populations relative to the effects generated by individual stressors. The results of multiple‐stressor studies have indicated additive and synergistic interactions between stressors and endocrine disruption, but this depends on the level of biological organisation included (Fischer, Pomati & Eggen, 2013; Sulmon *et al*., 2015). Consequently, such processes are significant in altering the observed effects of EDC exposure whilst also demonstrating the need for analyses to encapsulate the effects of ecological processes on sub‐lethal EDC impacts.

### (6) Effects of population genetics on responses to EDC exposure

Interactions between the wider spatial connectivity of aquatic environments (e.g. isolated and connected populations) and chemical contamination can have marked effects on the genetic diversity present within populations (Bickham *et al*., 2000). Genetics, specifically genetic diversity, can play an important role in determining the effects of EDC exposure, with reductions in genetic diversity derived from inbreeding potentially increasing the adverse ecological effects of EDC exposure (Bickley *et al*., 2013). Söffker, Stevens & Tyler (2012) reported that despite a generally similar response of genetically divergent *D. rerio* populations to EE2 exposure, differences in their breeding biology and response sensitivity were apparent. Inbreeding within laboratory fish stocks is a major issue for experimental assessments of EDCs, especially when intending to inform further research in systems involving outbred individuals (Brown *et al*., 2009). Although perhaps of limited value for building understanding of the effects of EDCs in outbred populations, experimental bioassays using inbred individuals may be useful for indicating the increased susceptibility of isolated natural populations to EDC exposure. In the event of habitat reconnection, whereby inbred and outbred populations interact, adverse impacts on fertility within inbred populations can facilitate a reduction in reproductive output of inbred individuals (Bickley *et al*., 2013). Assessments analysing interactions between genetic diversity and endocrine disruption within natural populations however remain scarce, and future research is required to test several hypotheses relating to genetic diversity and endocrine disruption across the wider aquatic environment.

### (7) Trophic cascades and other indirect effects of EDCs

Direct effects of endocrine disruption may cause alterations in processes and interactions within aquatic ecosystems, in turn generating indirect effects across wider levels of biological organisation (Relyea & Hoverman, 2006; Schulz *et al*., 2015). Such secondary effects may result from changes in competition and predation interactions within food webs, and subsequent release from biotic stressors (Knight *et al*., 2005). Similar trophic cascades have been identified to result from other anthropogenic contaminants, such as petroleum hydrocarbons and heavy metals (Fleeger, Carman & Nisbet, 2003). Very few studies, however, have assessed these phenomena for EDCs. These indirect processes could alter the perceived impacts of EDC exposure within natural populations, as well as affect the transfer of EDCs within food webs. Indirect effects may occur through several mechanisms. Knapp *et al*. (2005) demonstrated that changes in nutrient fluxes resulting from invertebrate mortality in response to deltamethrin exposure (2000 ng l^−1^) increased microbial community biomass. A more commonly observed indirect mechanism is provided by the adverse effects of EDC exposure within predator assemblages and a subsequent top‐down cascade through the food web. Alterations in the structure of invertebrate communities have been recorded in response to failed recruitment of secondary‐consumer fish species when an entire Canadian lake was dosed with EE2 (5–6 ng l^−1^) over a period of three summers (Kidd *et al*., 2014). A similar example exists in a differently structured ecosystem, with endocrine disruption in *R. rutilus* populations resulting in a reduction in predation of phytoplankton and increased copepod abundance (Hallgren *et al*., 2014). The indirect effects of endocrine disruption and their influence over multiple trophic levels further indicates the potential for the observed effects of EDC exposure within natural systems to deviate from those predicted from experimental laboratory bioassays.

## IV. LIMITATIONS OF EDC IMPACT ASSESSMENTS

The results of assessments of the impacts of EDCs at broad spatial and temporal scales depart significantly from predictions from laboratory‐based experimental studies. These results highlight: (*i*) the limitations of using individual‐based bioassays to predict the effects of EDCs at population‐ and food‐web scales (also see Forbes *et al*., 2010; Hommen *et al*., 2010), and (*ii*) the need for research at a range of spatial and temporal scales to advance knowledge of broad‐scale ecological effects and risk assessment. The restricted scope of common experimental assessments has been highlighted previously (Matthiessen, 2008; Lecomte *et al*., 2013), with calls for additional data to inform existing protocols and enhanced higher‐tier tests to replace unsuitable testing methods (Taenzler *et al*., 2007). Although frameworks such as the OECD guidelines promote an increase in the complexity of assessments (Gourmelon & Ahtiainen, 2007), the methodologies used in these assessments inherently simplify the large range of controls on the effects of EDCs present within natural systems. Population‐level interactions, including density‐dependent relationships such as intra‐specific competition, provide inherent controls on the effects of EDC exposure within the environment, yet these controls remain absent from ecological impact and risk assessments (Mills & Chichester, 2005). The low ecological complexity inherent in these protocols therefore appears to provide a major constraint on the accuracy and wider applicability of such tests.

Models developed from standard, individual‐based bioassay protocols currently provide limited value for the investigation of the effects of EDCs within natural systems. As identified by Hazlerigg *et al*. (2014), isolation of the effects of chemical mediation from other sub‐lethal effects may underlie the underestimation of population‐level impacts in model scenarios. Although population‐level models are suggested as a method for generating environmentally relevant predictions across natural systems (Forbes, Calow & Sibly, 2008; Forbes *et al*., 2010, 2011), extrapolating from overly simplified experimental data must be done with caution. Furthermore, the availability of limited data at higher levels of biological organisation (e.g. populations) restricts the validation of model simulations (Rose *et al*., 1999; Forbes *et al*., 2008; Raimondo *et al*., 2009). The application of these models to the prediction of EDC effects across aquatic environments thus remains prone to inaccuracies (Munns *et al*., 2008).

## V. THE NEED FOR MULTI‐TIER INTEGRATED RESEARCH FOR STUDIES ON EDCS

Low environmental concentrations of EDCs, coupled with their high propensity for sub‐lethal impacts, means that assessments at broader scales are essential for understanding the true implications of EDC exposure. Nonetheless, the complex mechanisms through which endocrine disruption can occur requires a detailed causal understanding which is difficult to derive from large‐scale studies (e.g. mesocosm or field assessment) (Schindler, 1998; Forbes *et al*., 2010). The requirement for a multi‐tiered research strategy may apply to all chemicals, but is arguably most relevant to EDCs due to their wide range of sub‐lethal effects that operate at different ecological scales, together with their potential for multiple biotic and abiotic interactions within and among spatial and temporal scales. The need to develop a cohesive, broad‐scale biomonitoring strategy is frequently identified in reviews of ecotoxicological risk assessments (Besse, Geffard & Coquery, 2012; Gavrilescu *et al*., 2015).

**Figure 2:**
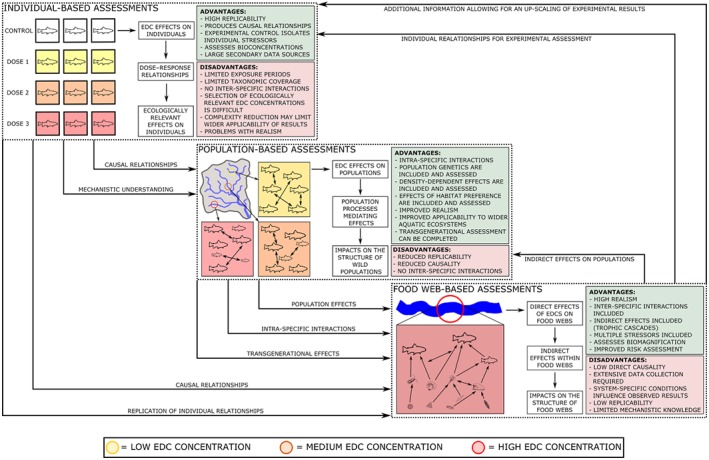
Interrelationships and information flow between micro‐, meso‐ and macroscale investigations for the biological impact assessment of endocrine‐disrupting chemical (EDC) exposure across a range of levels of biological organisation. Solid arrows indicate transfer of knowledge.

Knowledge acquired at multiple spatial and temporal scales provides a suitable framework to mitigate previous limitations and to increase our understanding of EDC effects over wider ecological scales. Similar integrated research has proved effective when assessing the complex effects of stressors within a range of ecosystems, including multiple stressors in freshwater systems (Altshuler *et al*., 2011) and heavy metals in coastal areas (Vlahogianni *et al*., 2007). In the case of endocrine disruption, such a focus will enable an increase in mechanistic knowledge at broad scales and the development of environmentally relevant experimental bioassays (Fig. 2). The product of this framework is environmentally relevant knowledge at a range of scales, enabling the provision of suitable information (and uncertainties) to practitioners and managers, potentially facilitating a reduction in adverse EDC effects across aquatic environments.

As in other research fields (see Culp *et al*., 2000), experiments on individuals can initially be used to understand the direct impacts of stressors at the organism level, and these can then be translated to research designs operating at broader scales. The multi‐tiered research strategy that we propose here, unlike other more‐specific ecosystem‐based strategies, is applicable to a wide range of ecosystems and a suite of EDCs. Furthermore, it surpasses previous methodological designs which focus more on the identification of ecological risk (using experimental bioassays) and subsequent biomonitoring programs (e.g. Maruya *et al*., 2013), rather than providing a framework for understanding the risks within all levels of biological organisation across ecosystems. Microcosm assessments within this research strategy allow for an assessment of EDC exposure on reproductive morphology, physiology and behaviour, in turn allowing for mechanistic knowledge at the organism and sub‐organism scales. Similarities and discrepancies identified between individual‐ and population‐level assessments can in turn indicate the population‐level processes and controls (e.g. density dependence and habitat‐mediated exposure) influencing the effects of EDCs within populations of aquatic organisms. Significant effects identified at the population level can be used to pinpoint areas of research suitable for further individual‐based studies. In terms of food‐web assessments, the initial direct effects identified within individual‐based assessments can indicate the potential for indirect effects and trophic cascades, allowing for the derivation of a suitable research design to identify these processes within natural systems. Furthermore, the high replicability and mechanistic understanding developed within individual‐based studies provides a valuable tool for broad‐scale assessment, enabling causal relationships to be derived for processes observed within aquatic food webs. The combination of individual‐, population‐ and food‐web‐level analyses can therefore enable improved realism of investigations, and facilitate up‐scaling of results to suitable levels for utilisation by practitioners.

## VI. FUTURE DIRECTIONS

### (1) Spatial variation in EDC concentrations across aquatic environments

Contemporary research focuses on up‐scaling EDC exposure to populations and food webs within aquatic environments. The spatial coverage of these assessments, however, is restricted when using individual systems to exemplify the wider conditions present across the landscape. An example of this is the focus on WwTWs and their downstream impacts across aquatic systems. A focus on wild populations and the effects of regulated effluent discharges (containing EDCs) has made significant contributions to establishing the effects of effluent discharges on aquatic organisms across aquatic environments. However, a focus on WwTWs discharges has also led to limitations in our understanding of the spatial variation in EDC occurrence and their impacts within and between different types of aquatic systems. Up‐scaling research strategies to landscape scales to understand these spatial variations is much needed to extend our knowledge of the effects of EDCs within natural systems. This will enable improved impact and risk assessment, with practitioners able to assess more accurately the degree to which potential concerns vary across the aquatic environment. Water‐quality data regarding WwTWs discharges are available in many countries, consequently high‐risk WwTWs can be targeted for regulation and remediation. A range of techniques are available to achieve this objective, including spatial and statistical modelling. Modelling at extremely broad scales has identified variations in emission of steroidal oestrogens between catchments, highlighting spatial variation in effects (Zhang *et al*., 2014). Furthermore, a significant role of mixing zones in determining the distribution of EDCs has been identified at high resolutions (∼500 m) (Pagsuyoin, Lung & Colosi, 2012). Assessments investigating intra‐catchment variation, along aquatic continuums and among systems, however, are scarce. Understanding how EDC concentrations and subsequent exposure varies at this scale is extremely important for River Basin Management strategies currently employed by water managers.

### (2) EDC transfers across food webs

A detailed understanding of the transfer of EDCs across entire aquatic food webs is not yet available, with studies predominantly focusing on bioaccumulation and biomagnification of EDCs within upper trophic levels (Berglund, Nyström & Larsson, 2005). Assessments aiming to evaluate entire food webs are generally restricted to a small range of organisms representing several trophic levels. Controls on food‐web organisation, such as environmental conditions, may significantly influence EDC bioaccumulation, biomagnification and effects, whilst a range of other biological factors also provide important regulatory impacts. The extent to which these factors enhance (or mitigate) the transfer of toxicants through food webs, however, remains relatively unknown. Moreover, although existing studies document relatively variable relationships between biological controls and bioaccumulation of different EDCs across aquatic food webs, explanations for such variability are absent. Future work is required to detail the specific pathways of accumulation and magnification throughout the lower trophic levels to understand the routes of dietary EDC exposure and biomagnification within higher trophic‐level organisms. The first stage will be identifying the role of biotic and EDC‐specific processes in controlling trophic transfers. Comprehensive biological‐trait databases for aquatic organisms, such as Tachet *et al*. (2010), provide a valuable resource for such work.

### (3) Validation of biomarkers for quantifying EDC effects

Biomarkers, used to identify endocrine disruption within individuals, are well established for a small number of taxa, e.g. fish (Ankley *et al*., 2009). Methods for other taxa have received less attention, and their utilisation and validation is relatively poorly developed (see Matozzo *et al*., 2008). A recent review identified a wide range of established and novel techniques for identifying endocrine disruption across environmental samples, yet there is an absence of suitable data for their validation (Kudłak *et al*., 2015). Furthermore, the relative accuracy of biomarker assessments is widely debated, with inconclusive results for some novel biomarker techniques. For example, the use of vitellogenin as a biomarker of endocrine disruption in an amphipod (*Gammarus fossarum* Fabricius) proved inconclusive as vitellogenin expression was shown to vary with unexplained environmental conditions (Jubeaux *et al*., 2012). The unknown, potentially pleiotropic, function of the vitellogenin gene within male invertebrates also may limit the application of this biomarker in the assessment of endocrine disruption (Jubeaux *et al*., 2012). Further development and validation of biomarkers specific to EDCs therefore remains an important challenge (Kudłak *et al*., 2015). Relating the severity of endocrine disruption (*via* biomarker assessments) to analytical quantification of environmental EDC concentrations (e.g. *via* gas chromatography mass spectrometry) is essential for advancing our understanding of endocrine disruption in natural systems. Such comparisons will allow evaluation of the robustness of biomarkers in assessing ecological risk from EDCs and stimulate the refinement of *in vivo* methods. The currently restricted focus on a few chemicals and organisms limits the ability of practitioners to utilise biomarkers for ecological risk assessment and environmental decision‐making (Hutchinson *et al*., 2006). Establishing a wider database of biomarkers for multiple species and EDCs is therefore an important future goal.

### (4) Applying genetics and modelling to broad‐scale analysis

A significant concern surrounding EDCs is the potential for impacts on the genetic structure of populations and thus on the integrity of wild populations (Coe *et al*., 2008). Genetic assessments within natural systems, including DNA microsatellite and single nucleotide polymorphism (SNP) analysis, and other sequencing methods, provide the potential to assess whether EDCs affect population structure *via* genomic pathways (e.g. Harris *et al*., 2011). Olmstead, Lindberg‐Livingston & Degitz (2010) reported EDC‐induced sex reversal identifiable from genetic polymorphisms within the western clawed frog (*Xenopus tropicalis* Gray). As well as allowing for broad‐scale analyses, these techniques enable a reduction in the previously large number of samples required for field‐based assessments to detect reproductive impacts and sex reversal at low EDC concentrations.

Up‐scaling research into the effects of EDCs also requires improved models for populations and food webs. One major constraint in currently available population models is the absence of suitable parameterisation and validation data at the population level collected using field assessments (Rose *et al*., 1999; Raimondo *et al*., 2009). Future models must also aim at an improved representation of the biotic and abiotic controls present within natural systems (Borgå *et al*., 2004). Complexity, nonetheless, does not always facilitate accuracy, and highly site‐specific, overly complex models may lack wider applicability (Miller *et al*., 2007). New model strategies, such as developed by Rose *et al*. (2003), provide the way forward for future models, with a nested structure allowing incorporation of a range of multi‐scalar data, and in turn generating model simulations which replicate well the natural conditions found within ecological systems. Such work will enable an amalgamation of laboratory and field‐based data, facilitating an understanding of causality and environmental relevance within future research.

## VII. CONCLUSIONS

(1) The ecological effects of EDCs are currently investigated by effects assessments on individuals employing only a small number of different organisms under controlled experimental conditions. The environmental relevance of these findings is likely to be limited. Spatially and temporally up‐scaling these investigations within the aquatic environment is therefore vital in developing environmentally relevant knowledge and to provide supporting data for practitioners to make accurate risk assessments. The hormonal, sub‐lethal implications of EDC exposure could lead to a range of emergent effects resulting from ecological interactions.

(2) We have highlighted the potential benefits of applying previously derived mechanistic knowledge at broader spatial and temporal scales to assess the ecological impacts of EDC exposure within natural systems. A range of abiotic and biotic characteristics and processes can alter the effects and transfer of EDCs within aquatic food webs and cause deviations of observed effects from those identified in experimental assessments. A range of indirect effects also occur within natural systems, thus accurate assessment of endocrine disruption risk within aquatic ecosystems requires an appreciation of ecological processes at a range of spatial and temporal scales.

(3) Several limitations of experimental bioassay designs are highlighted by recent research assessing broad‐scale EDC exposure. Consequently, the results of experimental bioassays should be interpreted with caution as such investigations often poorly represent influential controls present in natural systems. It is suggested that chemical test guidelines and models developed using these bioassays may provide limited utility in assessing the impacts and risk associated with EDCs.

(4) A complementary suite of assessments at a range of scales should be adopted within a multi‐tier integrated research strategy to promote the development of environmentally relevant knowledge suitable for use by practitioners. Understanding the various direct and indirect impacts of EDCs, across a range of different spatial and temporal scales, should allow us to determine more effectively the transfer and ecological effects of EDCs within natural systems. Increasing the effectiveness of empirical and experimental research through methods such as integrated frameworks is therefore an important development.

(5) Future research should focus on expanding field‐based research across a range of different aquatic environments. To achieve this objective, however, methodological and theoretical advances are required to enhance their applicability to natural systems and to develop more comprehensive methods of risk assessment for EDCs.

## VIII. ACKNOWLEDGEMENTS

This work was supported by the Natural Environmental Research Council [NE/L002434/] (F. M. W.). The authors would like to thank two anonymous reviewers for their comments.
